# H3K4me2 Promotes the Activation of lncCPSET1 by Jun in the Chicken PGC Formation

**DOI:** 10.3390/ani11061572

**Published:** 2021-05-27

**Authors:** Chen Zhang, Qisheng Zuo, Xiaomin Gao, Cai Hu, Shujian Zhou, Chen Chen, Yichen Zou, Juanjuan Zhao, Yani Zhang, Bichun Li

**Affiliations:** 1 Joint International Research Laboratory of Agriculture and Agri-Product Safety of Ministry of Education of China, Key Laboratory of Animal Breeding Reproduction and Molecular Design for Jiangsu Province, College of Animal Science and Technology, Yangzhou University, Yangzhou 225009, China; 18252774437@163.com (C.Z.); 006664@yzu.edu.cn (Q.Z.); g19895320723@163.com (X.G.); hucai18852729467@163.com (C.H.); zhoushujian1997@163.com (S.Z.); carolchen1996@163.com (C.C.); dachenzide@163.com (Y.Z.); zjj1632020@163.com (J.Z.); ynzhang@yzu.edu.cn (Y.Z.); 2 College of Biotechnology, Jiangsu University of Science and Technology, Zhenjiang 212000, China

**Keywords:** chicken, lncRNA, H3K4me2, DNA methylation, primordial germ cells

## Abstract

**Simple Summary:**

The formation of primordial germ cells is a knotty process involving both genetic and epigenetic factors. However, the exact mechanism is not clear. Here, we found a new lncRNA, which is highly expressed in the primordial germ cell. Histone H3K4 methylation could accelerate the formation of primordial germ cells by regulating this lncRNA. Our study provides a reference for exploring the formation mechanism of germ cells, breeding work, and the protection of endangered animals.

**Abstract:**

Primordial germ cells are the ancestors of female and male cells. Current research has shown that long non-coding RNA (lncRNA) and Histone methylation are the pivotal epigenetic factors in the PGC formation. However, there are few studies on the regulatory mechanism of lncRNA in the formation of PGC. Here, we define the lncRNA highly expressed in chicken PGC, lncCPSET1 (chicken-PGC-specifically-expressed transcript 1) This study found that compared with the interference of lncCPSET1/histone methylase Mll2 alone, the PGC formation was severely inhibited with the interference of lncCPSET1 and histone methylase Mll2 jointly in vivo and in vitro. Studies on the transcription level of lncCPSET1 found that H3K4me2 and transcription factor Jun have a positive effect on the activation of lncCPSET1; while DNA hypomethylation inhibits the expression of lncCPSET1. In terms of mechanism, compared with DNA methylation, H3K4me2 dominates lncCPSET1 activation. H3K4me2 can be enriched in the lncCPSET1 promoter, change its chromosome conformation, recruit the transcription factor Jun, and activate the expression of lncCPSET1. Taken together, we confirmed the model that H3K4me2 rather than DNA hypomethylation mediates Jun to regulate lncCPSET1 transcription, which broadens the study of lncCPSET1 pre-transcriptional mechanism.

## 1. Introduction

Primordial germ cells (PGC) are the ancestors of male and female germ cells, which occupy an indispensable position in the process of growth and development of the body. Bird PGC can migrate through the blood and settle to the gonadal ridge. Based on this feature, exogenous modified PGC was injected into the body and transgenic chickens carrying foreign genes were obtained, which is expected to realize the wide application of transgenic technology in birds [1,2,3]. However, due to the unclear research on the mechanism of PGC, it is inefficient to obtain PGC in vitro, which seriously limits the application of PGC-mediated transgenic birds’ production. In previous studies, scientists have tried to screen key genes or signal pathways [4,5,6] in the process of PGC formation to solve the difficulty of obtaining PGC from the perspective of genetics, but the progress was not obvious [7]. This suggests that we need to explore the formation mechanism of chicken PGC from another angle–epigenetic perspective.

More and more studies have confirmed that lncRNA is widely involved in various biological processes. LncRNA (Fendrr) [8] is essential in the development of mouse embryonic hearts and body walls. At the same time, lncRNA [9,10] can also maintain cell homeostasis through DNA damage repair and improve the efficiency of reprogramming fibroblasts into iPS [11]. Current studies focus more on the role of aberrant expression of lncRNA in carcinogenesis [12,13,14,15]. Different from the above, the research of lncRNA in the process of PGC formation has not been carried out systematically, and only a few studies have explored the expression pattern of lncRNA in PGC. Chen [16] found 38 kinds of lncRNA with high expression in PGCLC and low expression in embryonic stem cell (ESC) in mice. Bao [17] analyzed the sequencing of the development of male germline cells and found that lncRNA was dynamically expressed in PGC reprogramming, meiosis, and haploid et al. six stages, indicating that many lncRNAs were involved in the formation and occurrence of PGC. However, the functions of a lot of potential lncRNAs in the formation of PGC in poultry have not been explored.

In terms of mechanism, studies have shown that lncRNA participates in life activities in a variety of ways, mainly pre-transcriptional and post-transcriptional regulation. The current research focuses on the post-transcriptional regulation of lncRNA, including ceRNA mechanism [18], signal transduction mechanism [19], and recruitment protein mechanism [20]. However, there are few studies on lncRNA pre-transcriptional. It is well known that transcription factors and epigenetic modification can regulate gene expression, and the transcriptional regulation of lncRNA is similar to that of genes. Therefore, we speculate that transcription factors and epigenetic modifications (DNA methylation, histone methylation) also regulate the expression of lncRNA.

This study defined a lncRNA--lncCPSET1 (chicken PGC-specifically-expressed transcript 1) specifically expressed on PGC, which combined with H3K4me2 can effectively promote the formation of PGC. In-depth analysis, it was found that H3K4me2 was enriched in the promoter region of lncCPSET1, changed its chromosome conformation, and recruited transcription factor Jun, to activate the expression of lncRNA. Interestingly, although DNA methylation is also involved in the regulation of lncCPSET1 expression, the main factor for activating lncCPSET1 is H3K4me2 rather than DNA hypomethylation. Our results not only explored the regulation mechanism of key lncRNA expression in chicken PGC, but also further compared the role of different epigenetic factors in regulating lncRNA expression, providing a new perspective for improving the efficiency of PGC acquisition in vitro.

## 2. Materials and Methods

### 2.1. Antibody and Reagent

Eggs (3000) were obtained from the farm of the Poultry Research Institute (Chinese Academy of Agricultural Science, Yangzhou, China) Lsd1 and Mll2 knockdown vectors were constructed by Zhang and preserved in the lab [21].

Commercially available antibodies were used: anti-H3K4me2 (ab32356, 1:2000 for WB experiment,10 μg for CHIP-qPCR experiment), anti-histone H3 (ab1791, 1:2000 dilution for WB experiment), anti-DDX4 (ab13840), anti-c-JUN (sc-376488 X), goat anti-mouse IgG (ab6786), goat anti-rabbit IgG (ab6718) from abcam (Cambridge, UK), anti-β-actin (CW0096M, 1:1000 for WB experiment) from Kangwei Century (Beijing, China).

Recombinant human bone morphogenetic protein-4 (BMP4, 25200072) is from PROSPEC (East Brunswick, NJ, USA); Dulbecco’s modified Eagle medium (DMEM, 41965062), fetal bovine serum (FBS, 10100-147), sodium pyruvate (11360-070), chicken serum (16110-082), pancreatin (25200072), knockout DMEM (10829018) were from Gibco (Carlsbad, CA, USA); β-mercaptoethanol (M3148), leukemia inhibitory factor from mouse (mLIF, L5158), fibroblast growth factor-basic human (bFGF, F0291), stem cell human (SCF, S7901), L-glutamine (59202C), nonessential amino acids (M7145), conalbumin (C7786-100) from Sigma (Saint Louis, MO, USA), penicillin-streptomycin solution (P1400-100), 4% paraformaldehyde (P1110), Triton X-100 (T8200), HEPES (H1095) from Solarbio (Beijing, China). IL-11(200-11) from Peprotech (Rocky Hill, NJ, USA). FuGENE^®^ HD (E2311) from Promega (Madison, WI, USA); shRNA target sites sequence of genes are shown in Supplementary Table S1.

ESC Medium: Knockout DMEM + 10% FBS + 0.1 mmol/L β-mercaptoethanol +1 mmol/L sodium pyruvate + 2 mmol/L L-glutamine + 1% nonessential amino acids + 1000 IU/mLLIF + 10 ng/mL bFGF + 5 ng/mL SCF + 1% penicillin-streptomycin solution + 2% chicken serum.

ESC Induction Medium: DMEM + 1× nonessential amino acids + 0.1 mM β-mercaptoethanol + 1× penicillin-streptomycin solution + 1 mM sodium pyruvate + 10 ng/mL LIF + 40 ng/mL BMP4 + 50 ng/mL EGF.

PGC Medium: DMEM + 10% FBS + 2% chicken serum + 1 mM sodium pyruvate + 55 uM β-mercaptoethanol+ 20 ng/mL conalbumin + 10 mM HEPES + 1× penicillin-streptomycin solution + 5 ng/mL hSCF + 5 Units/mL mLIF + 10 ng/mL bFGF + 0.04 ng/mL IL-11.

### 2.2. Cell Isolation and Culture

ESC, PGC, and spermatogonial stem cell (SSC) were isolated separately from X-stage fertilized eggs, genital ridges, and testes. The cell isolation methods referred to the Zhang et al. [21]. ESC Induction Medium was used to induce ESC to PGC-like. The culture medium was replaced every 2 d. Cell morphology was observed by an inverted microscope and cell samples were collected for subsequent experiments.

### 2.3. Quantitative Reverse Transcription PCR (qRT-PCR)

Total RNA was extracted from tissues and cells using TRIzol Reagent (DP424, TIANGEN, Beijing, China) cDNA was synthesized by reverse transcription of Takara. qRT-PCR was performed according to the instructions by using SYBR in a fluorescent quantitative PCR kit and the 7500 Biosystems (Applied Biosystems, Carlsbad, CA, USA) to detect the related gene expression. β-actin was used as an internal control gene. The expression level of the relative genes was calculated by the formula 2−ΔΔCt. qRT-PCR primers are shown in Supplementary Table S2.

### 2.4. Flow Cytometry (FCM) Assessment

Samples were collected on the 6th day under BMP4 induction and labeled by DDX4. PGC was isolated from the 4.5th day embryo that was labeled by DDX4. FCM was used to detect the number of PGC.

### 2.5. Indirect Immunofluorescence

After induction, cells were fixed with 4% paraformaldehyde for 30 min, washed with PBS three times, and then permeabilized with 0.5% Triton X-100 for 10 min. Then, the slices were washed three times with PBS and sealed with 10% bovine serum albumin (BSA) in PBS for 30 min at room temperature. After that, the first antibodies against DDX4 were added, and the slices were incubated overnight at 4 °C. After washing three times with PBS-Tween (PBST), the corresponding second antibody was applied, and then incubated in darkness at 37 °C for 2 h. After washing three times with PBST, DAPI was used for nuclear staining. Fluorescence microscopy was used to observe the slices.

### 2.6. Luciferase Reporter Assays

The Dual-Luciferase Reporter Assay System was used to monitor the promoter activity. DF1 cells were cotransfected with 1000 ng plasmids of SV40 and respective reporter constructs (1:30) Firefly and Renilla luciferase activity were measured after 48 h using a plate reader (Winooski, Vermont, USA). The firefly luciferase signal was normalized against the Renilla signal and the empty vector PGL3-basic as control.

### 2.7. CHIP-qPCR

Collected cells and treated with formaldehyde to crosslink, then added SDS Lysis Buffer into the cells. Sonicated cell lysate on wet ice to shear DNA. Removed supernatant to fresh microfuge tubes in 100 uL aliquots. Then crosslinked protein/DNA were used to carry out the Immunoprecipitation experiment. After elution of protein/DNA complexes, protein/DNA complexes were reversed cross-linked to free DNA. Then used spin columns to Purify DNA. qRT-PCR was used to detect the enrichment of protein or H3K4me2. See Supplementary Table S3 for a list of primers for CHIP-qPCR.

### 2.8. Bisulfite Genomic Sequence (BSP)

Genomics was extracted from cells, and DNA was treated with a bisulfite kit (DP215 TIANGEN) Then carried out DNA amplification. The fragments were recovered and connected with the carrier. Carrier was used to transformant competent cells. After 12 h of culture, 10 colonies were selected for sequencing. Used the online website (http://quma.cdb.riken.jp/top/index.html. accessed on 15 May 2019) to make a pie chart. PCR Primer sequences see Supplementary Table S4.

### 2.9. Western Blot

Collected cells and added Lysis buffer PMSF (P0100, Solarbio): RIPA (CW2333, CWBIO, Beijing, China) = 1:100. BCA kit (CW0014, CWBIO) was used to detect the protein concentration. Equal amounts of protein were separated by sodium dodecyl sulfate-polyacrylamide gel electrophoresis (SDS-PAGE) and transferred to the polyvinylidene difluoride membrane. The membrane was blocked with 5% nonfat milk for 2 h and incubated with primary antibodies overnight at 4 °C, followed by secondary antibodies for 1 h at room temperature. Protein bands were detected using a BIO-RAD imaging system.

### 2.10. Statistical Analysis

Relative gene expression was calculated using the 2−ΔΔCt method after PCR. T-test analysis was performed using the SPSS 18.0 software (IBM, Armonk, NK, USA). All experiments were performed by three repetitions in biology and technology. The results are expressed as an average. (* *p* < 0.05, significant difference. ** *p* < 0.01, extremely significant difference) GraphPad Prism7 software (GraphPad Software, San Diego, CA, USA) was used for mapping.

## 3. Results

### 3.1. H3K4me2 Combined with lncCPSET1 Promoted the Formation of PGC In Vivo

In our previous study, 15 differentially expressed lncRNA related to germ cell differentiation in PGC were screened by RNA-seq sequencing [22]. The target genes of differential lncRNAs were predicted by cis, in which BMP4 is the key factor affecting the formation of PGC [23,24]. Therefore, this study focused on lncRNA TCONS_00874170 targeting BMP4 and named it lncCPSET1 (chicken-PGC-specifically-expressed transcript 1) qrt-PCR verified the sequencing results (Supplementary Figure S1A) Compared to the homology of lncCPSET1 transcripts by NONCODE, it was found that lncCPSET1 had low homology in different species, and NONHSA T257267.1 in human obtained the highest score (Supplementary Table S5) However, only 20 bases of NONHSA T257267 matched lncCPSET1 and this lncRNA can express in testis (Supplementary Figure S1B,C), indicating that lncCPSET1 is only highly conserved and unique in chickens.

**Figure 1:**
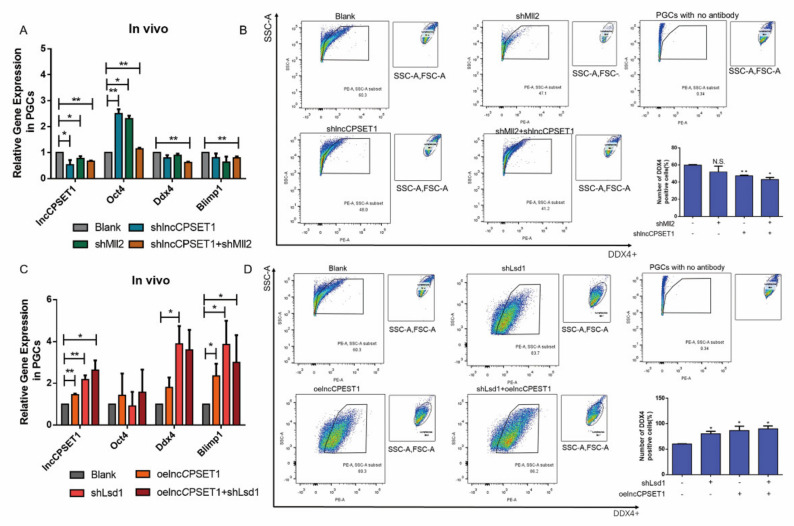
H3K4me2 combined with lncCPSET1 promotes the formation of PGC in vivo. (**A**) The relative mRNA expression of genes (lncCPSET1, Oct4, Ddx4, and Blimp1) after knockdown of lncCPSET1 and Mll2. (**B**) Flow cytometry analysis of DDX4+ positive cells was determined after knockdown of lncCPSET1 and Mll2. (**C**) The relative mRNA expression of genes (lncCPSET1, Oct4, Ddx4, and Blimp1) after knockdown of Lsd1 and overexpression of lncCPSET1. (**D**) Flow cytometry analysis of DDX4+ positive cells was determined after knockdown of Lsd1 and overexpression of lncCPSET1. The experiments were repeated three times. Error bars indicate the means ± S.E.M. * *p* < 0.05, ** *p* < 0.01. 3.2 H3K4me2 combined with lncCPSET1 promoted the formation of PGC-like in vitro.

Previous studies have found that lncCPSET1 can promote the formation of PGC (data not shown), and H3K4me2 has also been proved to be a key epigenetic factor in the formation of PGC1. Therefore, this study committed to exploring the effect of H3K4me2 on the regulation of lncCPSET1 in PGC formation. The lentivirus interference vectors of lncCPSET1 and Mll2 were simultaneously injected into chicken embryos. qRT-PCR showed that interfering with lncCPSET1/Mll2 alone, the expression of lncCPSET1 significantly decreased and totipotent marker Oct4 increased (*p* < 0.05) Interfered with Mll2 and lncCPSET1 simultaneously, the expression of lncCPSET1 and reproductive marker genes Ddx4 and Blmp1 were extremely significantly reduced (*p* < 0.01), Oct4 is extremely significantly increased (*p* < 0.01; Figure 1A) We have detected the specific expression of DDX4 antibody in chicken PGC previously [25]. Here, flow cytometry showed that the number of DDX4+ cells decreased after interfering with Mll2 or lncCPSET1. After interfering with Mll2 and lncCPSET1 simultaneously, the number of DDX4+ cells significantly decreased (*p* < 0.05; Figure 1B) On the contrary, the number of PGC increased significantly after interference with Lsd1 and overexpression of lncCPSET1 detected by qRT-PCR and flow cytometry (*p* < 0.05; Figure 1C,D) The above results suggested that lncCPSET1 is a positive regulator of PGC formation, and H3K4me2 enhances the ability of lncCPSET1 to promote PGC formation.

### 3.2. H3K4me2 Combined with lncCPSET1 Promoted the Formation of PGC-Like In Vitro

**Figure 2:**
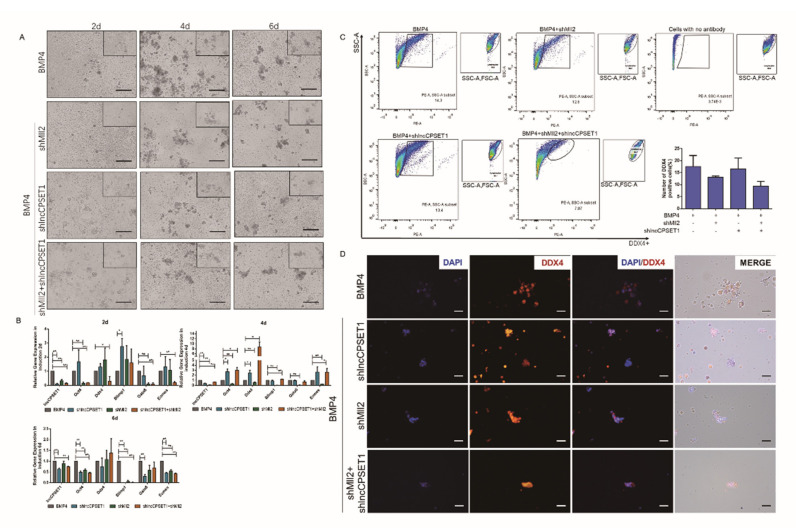
Knockdown of Mll2 and lncCPSET1 inhibits PGCLC formation in vitro. (**A**) Cell morphology was observed at different days of induction (2d, 4d, and 6d) under knockdown of Mll2 and lncCPSET1. (**B**) The relative mRNA expression of genes (lncCPSET1, Oct4, Ddx4, Blimp1, Gata6, and Ecomes) after knockdown of lncCPSET1 and Mll2 in different induction days (2d, 4d, and 6d) (**C**) Flow cytometry analysis of DDX4+ positive cells was determined after knockdown of lncCPSET1 and Mll2 in 6d of BMP4 induction. (**D**) Indirect immunofluorescence showed the DDX4+ positive cells in different groups in 6d of BMP4 induction. The experiments were repeated three times. Error bars indicate the means ± S.E.M. * *p* < 0.05, ** *p* < 0.01.

This study also used the BMP4 induction model in vitro to explore the regulation of H3K4me2 combined with lncCPSET1 in PGC formation. Morphological observation showed that embryoids appeared on the 4th day with BMP4. The volume of embryoids increased and a large number of clusters appeared subsequently. Meanwhile, the quantity of embryoid body decreased and the cell distribution is more scattered with the interference of Mll2 or lncCPSET1. What’s more, the number of embryoid bodies still decreased after interference with Mll2 and lncCPSET1 simultaneously (Figure 2A) Studies have shown that during the induction of ESC to PGC-like, ESC will first be induced into the embryoid body, and cells, which can express PGC marker genes, derived from the embryoid body are PGC-like. In this study, the expression of lncCPSET1 and PGC marker gene Blimp1 significantly decreased after interfering with Mll2 on the 4th day by qRT-PCR. Also, the expression of lncCPSET1, Blimp1, and germ layer differentiation-related genes Gata6 and Eomes decreased significantly whether interfered with Mll2 or lncCPSET1 separately or simultaneously (*p* < 0.01; Figure 2B) Flow cytometry illustrated that the proportion of positive cells decreased after interfering with Mll2 or lncCPSET1, while cells reached the lowest number when interfering with Mll2 and lncCPSET1 simultaneously on the 6th day (Figure 2C) Interfering with Mll2 and lncCPSET1 simultaneously inhibited the DDX4 positive cells (Figure 2D).

On the contrary, after interfering with Lsd1 and overexpressing lncCPSET1, the number of PGC-like increased (Supplementary Figure S2A–D) The above results indicated that H3K4me2 promotes the regulation of lncCPSET1 to the formation of PGC-like in vitro.

### 3.3. Double Epigenetic Regulation of lncCPSET1 Expression by H3K4me2 and DNA Methylation

**Figure 3:**
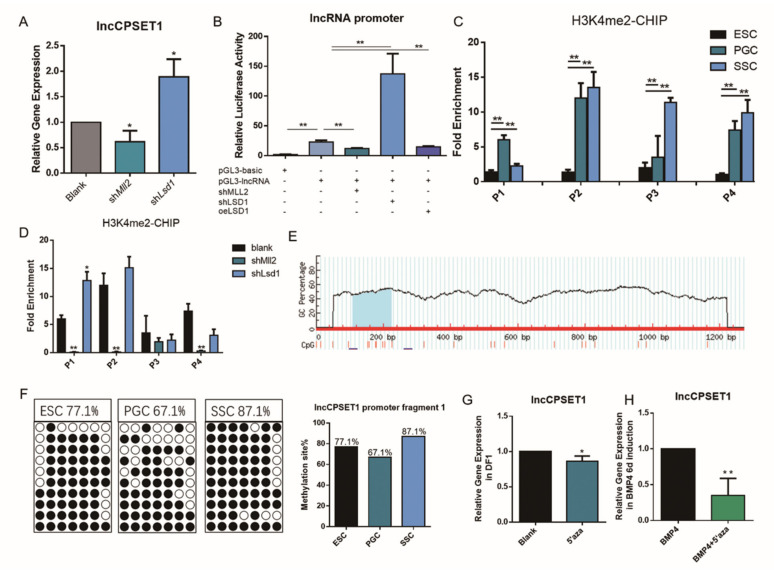
Dual epigenetics (H3k4me2 and DNA methylation) regulate the expression of lncCPSET1. (**A**) The relative mRNA expression of lncCPSET1 after knockdown of Mll2 and Lsd1 in PGC. (**B**) The initiation activity of lncCPSET1 was inhibited after interference with Mll2 and was enhanced after interference with Lsd1 by luciferase reporter assays. (**C**) CHIP-qPCR assay revealed the fold enrichment of H3K4me2 in lncCPSET1 promoter in different cells (ESC, PGC, and SSC) (**D**) CHIP-qPCR assay was used to detect the H3K4me2 fold enrichment after knockdown of Mll2 or Lsd1 in lncCPSET1 promoter. (**E**) CPG island prediction in the promoter region of lncCPSET1. (**F**) The dot plot shows the DNA methylation level of the lncCPSET1 promoter region in three cells (ESC, PGC, and SSC) by bisulfite sequencing. (**G**) The relative mRNA expression of lncCPSET1 treated with 5′aza in DF1. (**H**) The relative mRNA expression of lncCPSET1 treated with 5′aza in BMP4 6d induction. The experiments were repeated three times. Error bars indicate the means ± S.E.M. * *p* < 0.05, ** *p* < 0.01.

In the above study we found that whether in vivo or in vitro induction process, H3K4me2 can affect the expression of lncCPSET1. Similar results were found in PGC (*p* < 0.05; Figure 3A) To explore the transcriptional regulation of lncCPSET1 by H3K4me2, double luciferase assay revealed that the activity of lncCPSET1 promoter decreased significantly after interfering with Mll2, while it increased significantly after interfering with Lsd1. With overexpression of Lsd1, the initiating activity of lncCPSET1 decreased significantly (*p* < 0.01; Figure 3B) The above results suggested that the regulation of lncCPSET1 expression by Mll2/Lsd1-mediated H3K4me2 on the transcriptional level mainly depends on the change of its initiating activity.

In order to show the H3K4me2 enrichment in the promoter of lncCPSET1, we carried out the CHIP-qPCR. Four pairs of quantitative primers (P1, P2, P3, P4) were designed according to the promoter region of lncCPSET1. CHIP-qPCR showed that the H3K4me2 enrichment of P1 in PGC was extremely higher than that in ESC (*p* < 0.01) Additionally, H3K4me2 enrichment in P2, P3, and P4 was mostly in SSC, which was extremely higher than that in ESC (*p* < 0.01; Figure 3C) The enrichment of H3K4me2 on the P1 fragment decreased extremely after interfering with Mll2 (*p* < 0.01), while that increased significantly after interfering with Lsd1 (*p* < 0.05) H3K4me2 enrichment in the P2 fragment had no change with the interference of Lsd1. The enrichment of H3K4me2 on the P3 fragment was not affected by histone methylase. On the P4 fragment, H3K4me2 enrichment had no change with the interference of Lsd1 (Figure 3D) The above results indicated that H3K4me2 enriched in the promoter P1 fragment of lncCPSET1, regulated by Mll2/Lsd1.

In addition to H3K4me2, other factors also could regulate the expression of lncCPSET1. Here, we found that there is a 199 bp-length CpG island in the P1 fragment of the lncCPSET1 promoter, which contains seven methylation sites (Figure 3E) Bisulfite sequencing revealed that 77.1% of the seven methylation sites were methylated in ESC, 67.1% of that methylated in PGC, and 87.1% of that methylated in SSC (Figure 3F) The expression of lncCPSET1 decreased significantly with DNA methylase inhibitor 5′aza by qRT-PCR in DF1 (*p* < 0.05; Figure 3G) Similar results were showed in the induction of ESC to PGC (*p* < 0.01; Figure 3H) These results suggested that low DNA methylation inhibited the expression of lncCPSET1 during PGC formation. The initiation of lncCPSET1 is regulated by double epigenetic modifications (histone H3K4me2 and DNA methylation).

### 3.4. Transcription Factors Jun Are Enriched in the Promoter Region to Regulate the Expression of lncCPSET1

**Figure 4:**
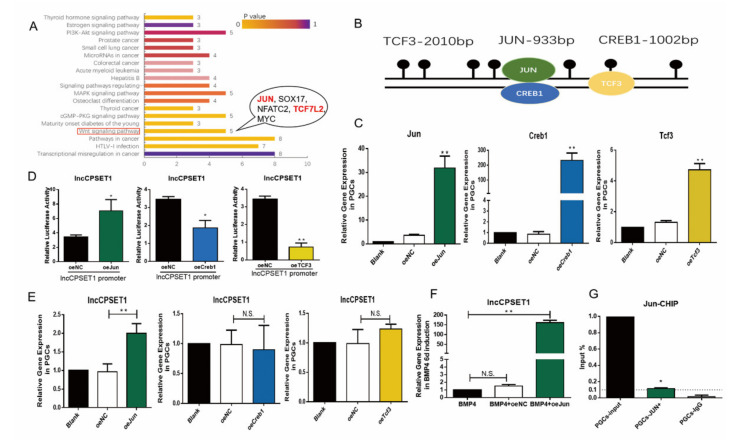
The transcription factor Jun is enriched in the promoter region to regulate the expression of lncCPSET1. (**A**) KEGG analysis showed the critical signaling pathway of transcription factor enrichment in the lncCPSET1 promoter. (**B**) The position and length of the three transcription factors on lncCPSET1 promoter. (**C**) qRT-PCR showed the corresponding gene expression after overexpression of transcription factor (Jun, Creb1, and Tcf3) in PGC. (**D**) The initiation activity of lncCPSET1 was detected by luciferase reporter assays after overexpression of transcription factor (Jun, Creb1, and Tcf3) (**E**) The relative mRNA expression of lncCPSET1 after overexpression of transcription factor (Jun, Creb1 and Tcf3) in PGC. (**F**) The relative mRNA expression of lncCPSET1 after overexpression of Jun in BMP4 6d induction. (**G**) CHIP-qPCR examined the enrichment of Jun in the lncCPSET1 promoter. The experiments were repeated three times. Error bars indicate the means ± S.E.M. * *p* < 0.05, ** *p* < 0.01.

Epigenetic modification often affects gene expression by changing chromatin conformation to regulate the binding of transcription factors to DNA. To find the potential transcription factor, in this study, transcription factors in the H3K4me2 and DNA methylation co-enrichment region P1 were predicted (JASPAR online website), with 79 potential transcription factors. GO annotation and KEGG enrichment analysis showed that transcription factors were enriched into 19 signal pathways, including PI3K-AKt, MAPK, Wnt, and other pathways involved in cell differentiation [26,27,28]. Three transcription factors, Jun, Tcf3, and Creb1, were selected for further study (Figure 4A) The overexpression vector of the transcription factor was successfully constructed and its activity was verified in DF1 and PGC (*p* < 0.01; Figure 4B,C, Supplementary Figure S3A–C) Double luciferase assay revealed that the initiating activity of lncCPSET1 increased significantly after overexpression of Jun, while that of lncCPSET1 decreased after overexpression of Creb1(*p* < 0.05) and Tcf3 (*p* < 0.01; Figure 4D) qRT-PCR showed that the expression of lncCPSET1 increased significantly with overexpression of Jun in DF1 and PGC (*p* < 0.05; Figure 4E, Supplementary Figure S3D–F) Similar results were obtained in the BMP4 induction model (*p* < 0.01; Figure 4F) Combined with CHIP-qPCR, it was found that JUN was significantly enriched in the P1 fragment of the lncCPSET1 promoter region (*p* < 0.05; Figure 4G) These results suggested that among three transcription factors, only Jun can enrich in the promoter region of lncCPSET1 to promote its expression in PGC formation.

### 3.5. H3K4me2 Promotes the Binding of Jun to lncCPSET1 Promoter to Activate Its Expression

**Figure 5:**
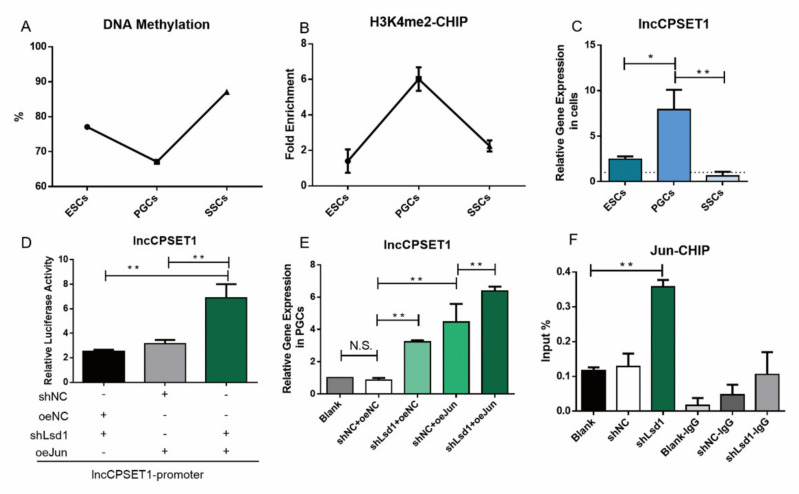
H3K4me2 promotes transcription factor Jun binding promoter region to activate the expression of lncCPSET1. A, B. Expression profile of DNA methylation (**A**) and H3K4me2 (**B**) in three cells (ESC, PGC, and SSC) (**C**) The relative mRNA expression of lncCPSET1 in three cells (ESC, PGC, and SSC) (**D**) The initiation activity of lncCPSET1 increased after knockdown of Lsd1 and overexpression of Jun. (**E**) The relative mRNA expression of lncCPSET1 in PGCs after knockdown of Lsd1 and overexpression of Jun. (**F**) CHIP-qPCR was used to detect the enrichment of Jun after knockdown of Lsd1. The experiments were repeated three times. Error bars indicate the means ± S.E.M. * *p* < 0.05, ** *p* < 0.01.

In the above study, we have found the expression patterns of DNA methylation, H3K4me2, and lncCPSET1 in three kinds of cells (Figure 5A–C), and DNA demethylation or H3K4me2 can regulate lncCPSET1. Here, this study explored the regulation of DNA demethylation or H3K4me2 to the activation of Jun on lncCPSET1. Double luciferase assay and qRT-PCR displayed that the initiating activity of lncCPSET1 significantly increased by interference with Lsd1 and overexpression of Jun simultaneously (*p* < 0.01; Figure 5D, Supplementary Figure S3G) Similar results were found in PGC (Figure 5E) CHIP-qPCR illustrated that the enrichment of Jun in the promoter of lncCPSET1 increased significantly after interfering with Lsd1 (*p* < 0.01; Figure 5F) Totally, H3K4me2 can promote the binding of Jun to the lncCPSET1 promoter and activate its expression.

### 3.6. DNA Hypomethylation Affects H3K4me2 Instead of Jun to Inhibit lncCPSET1 Expression

**Figure 6:**
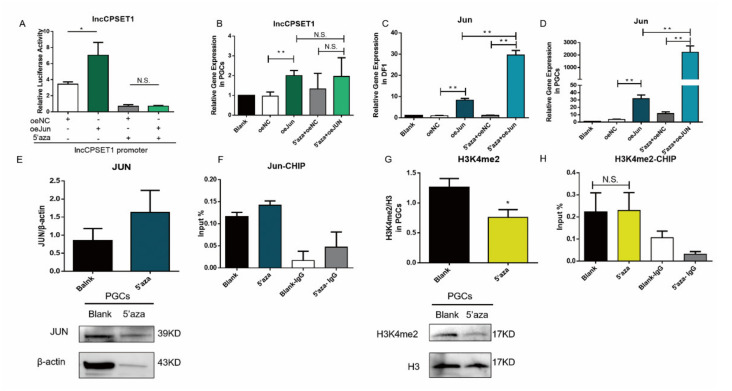
DNA hypomethylation affects H3K4me2 level instead of affects Jun level to inhibit lncCPSET1 expression. (**A**) The initiation activity of lncCPSET1 increased after overexpression of Jun and 5′aza treatment. (**B**) The relative mRNA expression of lncCPSET1 in PGC after overexpression of Jun and 5′aza treatment. (**C**,**D**) The relative mRNA expression of Jun in DF1 (**C**) and PGC (**D**) after overexpression of Jun and 5′aza treatment. (**E**) The protein expression of JUN was detected by western blot after 5′aza treatment in PGC. (**F**) The enrichment of Jun in the lncCPSET1 promoter was examined by CHIP-qPCR after 5′aza treatment in PGC. (**G**) The H3K4me2 level was detected by western blot after 5′aza treatment in PGC. (**H**) CHIP-qPCR was used to evidence the enrichment change of H3K4me2 in the lncCPSET1 promoter after 5′aza treatment. The experiments were repeated three times. Error bars indicate the means ± S.E.M. * *p* < 0.05, ** *p* < 0.01. Original western blot figures in Figure S4.

DNA hypomethylation inhibits lncCPSET1 initiation (Figure 3E–H) During the study of lncCPSET1, dual luciferase assay showed that the regulation of Jun on the lncCPSET1 promoter region significantly reduced with the decrease of DNA methylation (Figure 6A) qRT-PCR revealed that the expression of lncCPSET1 decreased with the treatment of 5′aza and overexpression of Jun simultaneously, compared with the overexpression of Jun alone (Figure 6B; Supplementary Figure S3H) It is suggested that the decrease of DNA methylation will inhibit the activation of Jun on lncCPSET1.

In order to clarifying the reason why DNA demethylation inhibits the lncCPSET1 transcription, this study puts forward two conjectures: (1) DNA demethylation inhibits the expression of transcription factors; (2) DNA demethylation affects the level of H3K4me2. Firstly, qRT-PCR displayed that the expression of Jun significantly increased with the treatment of 5′aza and overexpression of Jun in DF1 and PGC (*p* < 0.01; Figure 6C,D) The protein of JUN increased after the addition of 5‘aza, with no significant difference compared with blank by western blot (Figure 6E) CHIP-qPCR showed that the enrichment of Jun in the lncCPSET1 promoter increased after 5′aza treatment, with no significant change (Figure 6F) These results suggested that the inhibition of DNA hypomethylation on lncCPSET1 does not depend on reducing the expression or the binding ability of Jun.

Next, western blot revealed that the level of H3K4me2 significantly reduced with the treatment of 5′aza in PGC (*p* < 0.05; Figure 6G) CHIP-qPCR showed that the enrichment of H3K4me2 in lncCPSET1 promoter P1 had no significant change with the addition of 5′aza (Figure 6H) The above results demonstrated that the addition of 5′aza inhibited the expression of H3K4me2, and then reduced the lncCPSET1 expression.

### 3.7. H3K4me2 Is the Dominant Epigenetic Factor to Regulate the Activation of Jun to lncCPSET1 in PGC Formation

**Figure 7:**
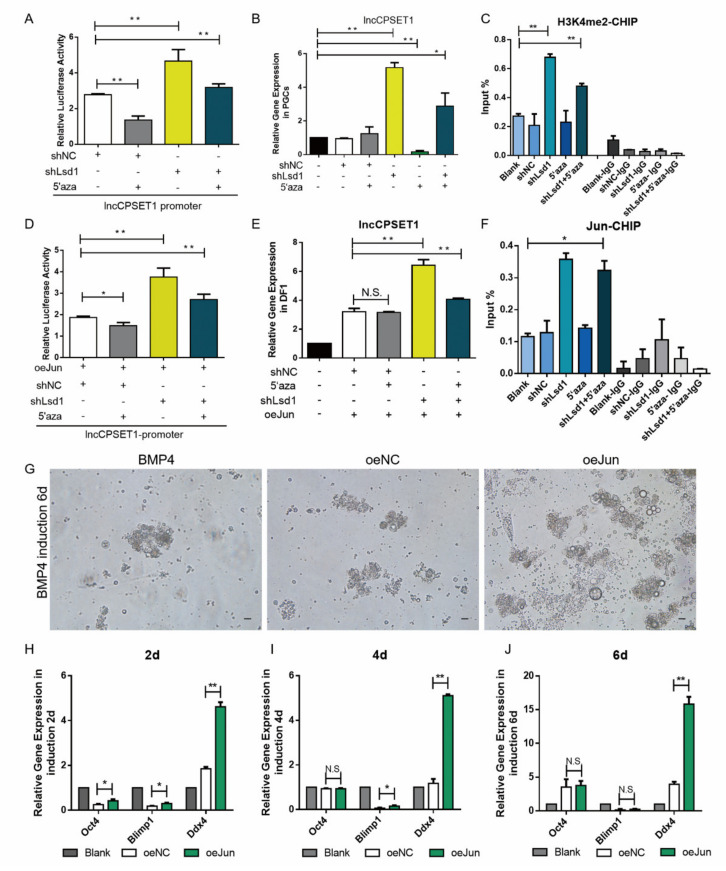
H3K4me2 participates in the formation of PGC as the dominant epigenetic factor regulating Jun activation of lncCPSET1. (**A**) The initiation activity of lncCPSET1 increased after the knockdown of Lsd1 and 5′aza treatment. (**B**) The relative mRNA expression of lncCPSET1 in PGC after knockdown of Lsd1 and 5′aza treatment. (**C**) CHIP-qPCR was used to evidence the enrichment change of H3K4me2 in the lncCPSET1 promoter after knockdown of Lsd1 and 5′aza treatment. (**D**) The initiation activity of lncCPSET1 increased after knockdown of Lsd1, overexpression of Jun and 5′aza treatment. (**E**) The relative mRNA expression of lncCPSET1 in DF1 after knockdown of Lsd1, overexpression of Jun and 5′aza treatment. (**F**) The enrichment of Jun in the lncCPSET1 promoter was examined by CHIP-qPCR after knockdown of Lsd1 and 5′aza treatment in PGC. (**G**) Cell morphology was observed at different days of induction (2d, 4d, and 6d) after overexpression of Jun. (**H**) The relative mRNA expression of Oct4, Blimp1, and Ddx4 in different induction days after overexpression of Jun. The experiments were repeated three times. Error bars indicate the means ± S.E.M. * *p* < 0.05, ** *p* < 0.01.

In order to exploring the effect of H3K4me2 and DNA hypomethylation on the expression of lncCPSET1, this study detected the initiating activity and expression of lncCPSET1. Double luciferase and qRT-PCR revealed that the initiating activity and expression of lncCPSET1 were significantly increased with the interference of Lsd1(*p* < 0.01), while that of lncCPSET1 decreased with the addition of 5′aza, which still increased significantly compared with control (*p* < 0.01; Figure 7A,B) Similar results were showed in PGC (Supplementary Figure S3I) CHIP- qPCR reported that despite the addition of 5′aza, H3K4me2 still could enrich in the lncCPSET1 promoter and the enrichment significantly increased with the interference with Lsd1 (*p* < 0.01; Figure 7C) The above results evidenced that DNA hypomethylation does not effect the enrichment of H3K4me2.

Next, the expression of lncCPSET1 was detected with the existence of H3K4me2, DNA hypomethylation, and transcription factor Jun. Despite the addition of 5′aza, the initiating activity of lncCPSET1 still significantly increased with the overexpression of Jun and interference of Lsd1 simultaneously (*p* < 0.01; Figure 7D) The results of qRT-PCR were consistent with that of the double luciferase assay (Figure 7E) CHIP- qPCR identified that the binding of Jun and lncCPSET1 promoter significantly improved with the interference of Lsd1 and the addition of 5′aza simultaneously (Figure 7F) The above results verified that H3K4me2 plays a leading role in regulating the combination of Jun and lncCPSET1 promoter.

Furthermore, this study explored the function of transcription factor Jun in the PGC formation. The number of embryoids significantly increased with the overexpression of Jun in vitro (Figure 7G) qRT-PCR showed that the expression of Ddx4 increased significantly in different induction stages (*p* < 0.01; Figure 7H–J) Flow cytometry revealed that the number of PGC-like increased on the 6th day after overexpression of Jun (Supplementary Figure S3J) The above results indicated the transcription factor Jun could promote the formation of PGC-like.

## 4. Discussion

Non-coding RNA plays an important role in a variety of biological processes, such as stem cell development, somatic differentiation, and carcinogenesis. A series of studies have confirmed that lncRNA participates widely in a variety of mechanisms, including signal, decoy, guide, and scaffold. However, there are few studies on the regulation of pretranscription epigenetic modification of lncRNA. Here, we reveal that a lncRNA--lncCPSET1, specifically expressed on chicken PGC, requires H3K4me2 rather than low DNA methylation-mediated transcription factor Jun activation in the transcriptional process.

The formation of PGC requires a variety of epigenetic modifications. Studies have shown that the formation of mouse PGC goes through the bivalent modification process of H3K9me2 and H3K27me3, and they are complementary and dynamically expressed. At the same time, in the formation of mouse PGC-like, ESCs reorganize their methylome, especially around pluripotency regulators, by de novo DNA methylation and H3K27ac removal, to form EpiLCs. Then the PGCLCs essentially dilute the EpiLC methylome and deposit H3K27me3 around developmental regulators for their repression [29,30]. Chen [16] sequenced the mouse PGC-like cells induced by ESC and found 38 specifically expressed lncRNA in PGC-like, which suggested lncRNA could be involved in PGC formation. The researchers also found that with DNA demethylation, PGC marker genes were activated while somatic marker genes were inhibited with histone methylation [31]. These studies suggested that multiple epigenetic modifications were participated in the PGC formation. However, there are few studies on the coordinated regulation of multiple epigenetic modifications in PGC formation. In this study, using chicken as a model, it was found that lncCPSET1 could promote the formation of PGC, while H3K4me2 could activate lncCPSET1. It is worth mentioning that under the combined action of the two, the efficiency of PGC formation in vivo and in vitro can be significantly improved. It shows that single epigenetic modification cannot meet the needs of complex PGC formation mechanisms, and the dual epigenetic modification of histone methylation and non-coding RNA plays an important role in the biological process.

Studies have shown that although lncRNA is poorly conserved among species, it has high tissue specificity. The pre-transcriptional regulation of LncRNA is affected by many factors. From the transcriptional factor regulation, Xie [32] found that transcriptional factor TP63 binds to super-enhancers to regulate the high expression of LINC01503; from the DNA methylation regulation, HadjiF [33] found that the decreased methylation level of CpG island is the reason for the high expression of lncH19. We found that lncCPSET1 has a similar pre-transcriptional regulation pattern. Although the level of DNA methylation is down-regulated during the formation of PGC, it inhibits the transcription of lncCPSET1. On the contrary, the transcription factor Jun can effectively increase the expression of lncCPSET1. This is similar to the results of XieJJ’s study [32]. The transcription of lncRNA could be regulated by transcription factors. Not only that, we also found that H3K4me2 plays an important role in regulating the transcriptional activation of lncRNA. It can be seen that, similar to genes, there are two ways of pre-transcriptional regulation of lncRNA: heredity and epigenetic.

DNA methylation is often considered as a lever for the regulation of the expression of proto-oncogenes and tumor suppressor genes in cancer research. The inactivation of many genes is related to the methylation of CpG islands in the promoter region. When Panning [34] was studying the regulation of lncXist expression, it was found that DNA hypomethylation activates Xist expression and silences X-linked gene expression. This result suggests that the regulation of gene expression by DNA methylation may be achieved by changing the level of upstream factors. In our study, we found a similar biological phenomenon, the addition of DNA methylase inhibitor 5′aza, not only did not activate lncCPSET1, but inhibited its expression, which is different from the traditional DNA methylation regulation mode. One possibility is that DNA hypomethylation affects the expression of transcription factors, which indirectly inhibits the initiation of lncCPSET1. Therefore, we detected the expression and the enrichment of transcription factor JUN after the treatment of DNA hypomethylation, then found that DNA hypomethylation could activate the expression of JUN, which could not explain the reduction of lncCPSET1 after DNA hypomethylation; the other is that DNA hypomethylation affects epigenetic modification. Obviously, our study belongs to the latter, that is, DNA hypomethylation inhibits the expression of H3K4me2 and reduces the expression of lncCPSET1. However, Kondo [35] found that after 5′aza treatment, the methylation level of H3K4 increased, and the expression of tumor suppressor genes was activated in human colon adenocarcinoma cells. Although our results deviated from the findings of Kondo, this may be because the effect of DNA methylation on the level of epigenetic modification varies with species.

As the key epigenetic modification to regulate the activity of the promoter region, which plays a major role in transcriptional regulation, DNA methylation, or histone methylation? Studies have shown that the dominant regulatory roles of them in different biological processes are still controversial. Jackson [36] believes that the methylation of H3K9 recruits HP1 or its homologs to bind to it, while DNA methylase methylates DNA through binding to HP1. Therefore, histone methylation plays a major regulatory role. However, Fahrner and Fuks [37] believe that DNA demethylation directs histone methylation and is the main epigenetic regulator. In this study, from the point of view of lncCPSET1 transcriptional regulation, it was found that H3K4me2 rather than hypomethylation of DNA promotes the binding of transcription factor Jun to the promoter to activate the expression of lncCPSET1. This result preliminarily proves that H3K4me2 plays a leading role in the regulation of lncCPSET1 expression, but the regulation mode between histone methylation and DNA methylation needs to be further discovered from a macro point of view.

## 5. Conclusions

Here, we obtained the lncRNA highly expressed in chicken PGC based on pre-transcriptome sequencing and named it lncCPSET1 (chicken-PGC-specifically-expressed transcript 1) Although lncCPSET1 could promote the formation of PGC, the regulation is controlled by H3K4me2. The specific manifestation is that H3K4me2 can be enriched in the lncCPSET1 promoter, change its chromosome conformation, recruit the transcription factor Jun, and activate the expression of lncCPSET1. Interestingly, the initiation of lncCPSET1 is also regulated by DNA hypomethylation, but H3K4me2 still plays a leading role in activation. Taken together, we confirmed the model that H3K4me2 rather than DNA hypomethylation mediates Jun to regulate lncCPSET1 transcription, which broadens the study of lncCPSET1 pre-transcriptional mechanism.

## Data Availability

The data underlying this article are available in the article and in its online supplementary material.

## Supplementary Materials

The following are available online at https://www.mdpi.com/article/10.3390/ani11061572/s1, Figure S1: Conservative analysis of lncCPSET1, Figure S2: Knockdown of Lsd1 and overexpression of lncCPSET1 promotes PGCLC formation in vitro, Figure S3: H3K4me2 dominates the expression of lncCPSET1, Figure S4: Original western blot figures for Figure 6 (E,G), Table S1: shRNA target sites sequence of genes, Table S2: qRT-PCR Primer sequences, Table S3: CHIP-qPCR Primer sequences, Table S4: PCR Primer sequences, Table S5: The conservative analysis of lncCPSET1.
